# Editorial: Clinical Application of Artificial Intelligence in Emergency and Critical Care Medicine, Volume II

**DOI:** 10.3389/fmed.2022.910163

**Published:** 2022-05-06

**Authors:** Zhongheng Zhang, Rahul Kashyap, Nan Liu, Longxiang Su, Qinghe Meng

**Affiliations:** ^1^Department of Emergency Medicine, Sir Run Run Shaw Hospital, Zhejiang University School of Medicine, Hangzhou, China; ^2^Critical Care Independent Multidisciplinary Program, Mayo Clinic, Rochester, MN, United States; ^3^Department of Anesthesiology and Perioperative Medicine, Mayo Clinic, Rochester, MN, United States; ^4^Programme in Health Services and Systems Research, Duke-National University of Singapore Medical School, Singapore, Singapore; ^5^State Key Laboratory of Complex Severe and Rare Diseases, Department of Critical Care Medicine, Peking Union Medical College Hospital, Chinese Academy of Medical Science, Peking Union Medical College, Beijing, China; ^6^Department of Surgery, SUNY Upstate Medical University, Syracuse, NY, United States

**Keywords:** artificial intelligence, critical care, emergency and critical care, heterogeneity, prediction model

We are excited to witness the successful publication of the second volume of the topic issue entitled *Clinical Application of Artificial Intelligence in Emergency and Critical Care Medicine*. Artificial intelligence (AI) continues to be a hot spot in emergency and critical care medicine, which provides numerous tools and algorithms to learn from data (1, 2). There are three major categories of machine learning (ML) algorithms including supervised learning (3), unsupervised learning, and reinforcement learning (4). These three main categories of ML methods can be utilized to solve clinical questions that cannot be well captured by human intuition.

Supervised learning is defined by its use of labeled datasets to train algorithms to classify data or predict outcomes accurately. As input data is fed into the model, it adjusts its weights until the model has been fitted appropriately, which occurs as part of the cross-validation process (5). The labels in the critical care setting include mortality, the occurrence of postoperative acute kidney injury, delirium, and the development of CRBSI (6–8). In this topic issue, several studies utilized a supervised ML algorithm to predict interested clinical outcomes. Cheng et al. developed a deep learning algorithm (ResNet50-V2) for the detection of abdominal free fluid in Morison's pouch during focused assessment with sonography in trauma. While the model is not externally validated, such seminal attempts will trigger further explorations on the use of radiomics in a critical care setting. Li et al. developed an ML algorithm using eXtreme Gradient Boosting (XGBoost) to predict acute renal failure after acute aortic syndrome surgery. The authors further demonstrated that the XGBoost algorithm was superior to the conventional logistic regression models.

Unlike specialty areas where patients are usually homogenous and thus can benefit from evidence-based medicine (EBM). Under the paradigm of EBM, a group of homogenous population are studied as a whole, and the effects drawn from the population are assumed to be equal in each individual subject (9). In other words, the individual effects are equal to the population mean. In such an ideal situation, a large well-conducted randomized controlled trial can provide the most effective treatment options for the target population. However, the individual effects deviate from the population mean with increasing heterogeneity of the study population. This is what happens in the critical care setting. Although critically ill patients are usually presenting similar clinical manifestations which we group as syndromes such as sepsis and acute respiratory distress syndrome (ARDS), these syndromes usually display significant heterogeneity in their clinical presentations and responses to treatment (10, 11). By a *post-hoc* analysis of a large ARDS trial, Xing et al. identified four phenotypes of ARDS, and the “hyperinflammatory Anasarca” phenotype was found to benefit from a conservative fluid management strategy. The strength of the study was the utility of a randomized trial. The randomization procedure ensured the exchangeability of the study population between treated and untreated groups. Latent class analysis was performed on an ARDS population, which identified three subphenotypes. A subtype characterized by worse renal function and higher central venous pressure (CVP) was found to be beneficial from diuretic use. Collectively, these studies demonstrated the successful application of the ML algorithm to identify subtypes of a heterogeneous population. Interestingly, these subtypes show both prognostic and predictive enrichment for clinical interventions.

The above-mentioned ML algorithms can only be helpful in risk stratification. We know a patient is at risk of death, but do not know how to take action to minimize the risks. In this regard, the third important algorithm called reinforcement learning comes to solve this clinical question (12, 13). Reinforcement learning is a type of machine learning technique where a computer agent learns to perform a task through repeated trial and error interactions with a dynamic environment. This learning approach enables the agent to make a series of decisions that maximize a reward metric for the task without human intervention and without being explicitly programmed to achieve the task. Fluid treatment is an important measure to improve the survival outcome of sepsis. However, the major challenge in fluid treatment is that it requires sequential decision rules to be made by considering the changing status of the subject, as well as its response to previous fluid challenges. Thus, there is no one-size-fits-all model for fluid therapy for sepsis patients. In this regard, Su et al. developed a reinforcement learning algorithm to optimize fluid treatment strategy. They further demonstrated that patients who received the fluid volume close to the estimated volume had the lowest mortality rate. However, since the algorithm is not tested in prospective studies, its clinical utility is still limited.

In conclusion, the topic issues on *Clinical Application of Artificial Intelligence in Emergency and Critical Care Medicine* witnessed tremendous success in the previous two volumes, suggesting continuous interest in this topic in critical care society. Volume III of the topic issue is still open for submission. With the developments in AI technology, it will change the practice pattern of emergency and critical care in the future.

## Author Contributions

ZZ conceived the idea and drafted the manuscript. NL, RK, and QM critically reviewed the paper and interpretation of the work. LS provided insightful comments for the work. All authors contributed to the article and approved the submitted version.

## Conflict of Interest

The authors declare that the research was conducted in the absence of any commercial or financial relationships that could be construed as a potential conflict of interest.

## Publisher's Note

All claims expressed in this article are solely those of the authors and do not necessarily represent those of their affiliated organizations, or those of the publisher, the editors and the reviewers. Any product that may be evaluated in this article, or claim that may be made by its manufacturer, is not guaranteed or endorsed by the publisher.
